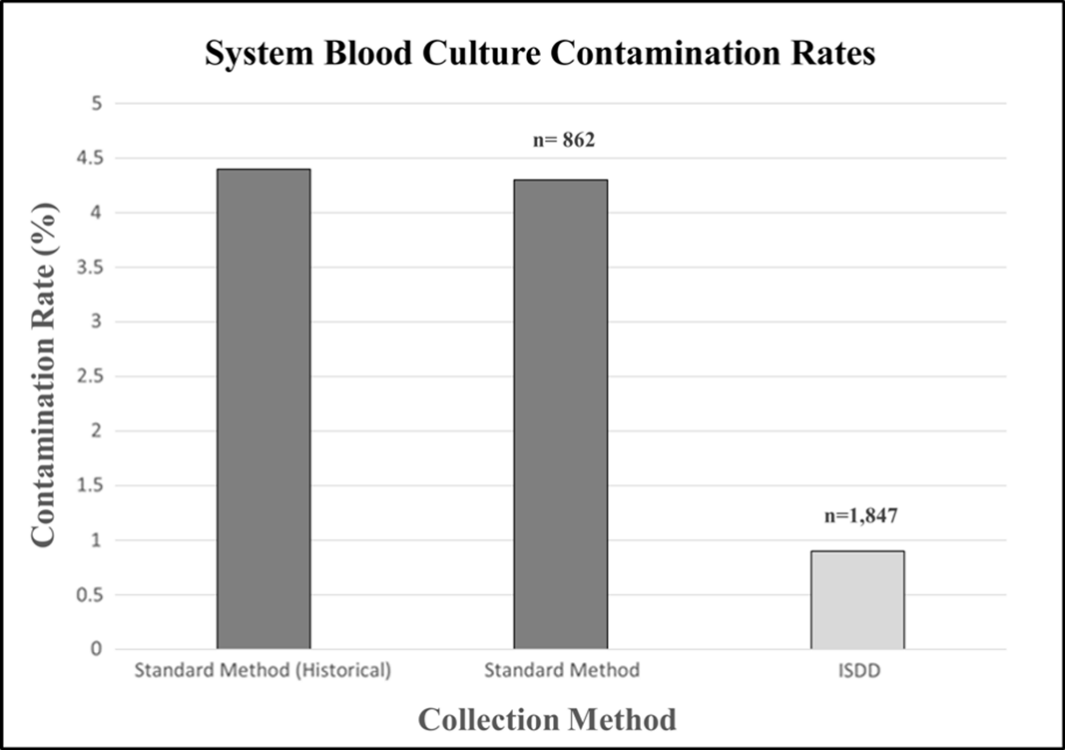# Antimicrobial Stewardship Standards and Patient Safety: A Case Study in Blood Culture Contamination

**DOI:** 10.1017/ash.2021.66

**Published:** 2021-07-29

**Authors:** Connie Schaefer

## Abstract

**Background:** Blood culture is a crucial diagnostic tool for healthcare systems, but false-positive results drain clinical resources, imperil patients with an increased length of stay (and associated hospital-acquired infection risk), and undermine global health initiatives when broad-spectrum antibiotics are administered unnecessarily. Considering emerging technologies that mitigate human error factors, we questioned historically acceptable rates of blood culture contamination, which prompted a need to promote and trial these technologies further. In a 3-month trial, 3 emergency departments in a midwestern healthcare system utilized an initial specimen diversion device (ISDD) to draw blood cultures to bring their blood culture contamination rate (4.4% prior to intervention) below the 3% benchmark recommended by the Clinical & Laboratory Standards Institute. **Methods:** All emergency department nursing staff received operational training on the ISDD for blood culture sample acquisition. From June through August 2019, 1,847 blood cultures were drawn via the ISDD, and 862 were drawn via the standard method. **Results:** In total, 16 contamination events occurred when utilizing the ISDD (0.9%) and 37 contamination events occurred when utilizing the standard method (4.3%). ISDD utilization resulted in an 80% reduction in blood culture contamination from the rate of 4.4% rate held prior to intervention. **Conclusions:** A midwestern healthcare system experienced a dramatic reduction in blood culture contamination across 3 emergency departments while pilot testing an ISDD, conserving laboratory and therapeutic resources while minimizing patient exposure to unnecessary risks and procedures. If the results obtained here were sustained and the ISDD utilized for all blood culture draws, nearly 400 contamination events could be avoided annually in this system. Reducing unnecessary antibiotic use in this manner will lower rates of associated adverse events such as acute kidney injury and allergic reaction, which are possible topics for further investigation. The COVID-19 pandemic has recently highlighted both the importance of keeping hospital beds available and the rampant carelessness with which broad-spectrum antibiotics are administered (escalating the threat posed by multidrug-resistant organisms). As more ambitious healthcare benchmarks become attainable, promoting and adhering to higher standards for patient care will be critical to furthering an antimicrobial stewardship agenda and to reducing treatment inequity in the field.

**Funding:** No

**Disclosures:** None

**Figure 1.**